# Identification of the potential crucial genes in invasive ductal carcinoma using bioinformatics analysis

**DOI:** 10.18632/oncotarget.23239

**Published:** 2017-12-13

**Authors:** Chunguang Li, Liangtao Luo, Sheng Wei, Xiongbiao Wang

**Affiliations:** ^1^ Department of Oncological Surgery, Zhongnan Hospital of Wuhan University, Wuhan, Hubei, P. R. China; ^2^ Department of General Surgery, First Renmin Hospital, Tianmen, Hubei, P. R. China; ^3^ Department of General Surgery, Traditional Chinese Medicine Hospital, Xishui, Hubei, P. R. China; ^4^ Department of General Surgery, First Renmin Hospital, Yangxin, Hubei, P. R. China

**Keywords:** invasive ductal carcinoma, bioinformatics analysis, differentially expressed genes

## Abstract

Invasive ductal carcinoma (IDC) is a common histological type of breast cancer. The aim of this study was to identify the potential crucial genes associated with IDC and to provide valid biological information for further investigations. The gene expression profiles of GSE10780 which contained 42 histologically normal breast tissues and 143 IDC tissues were downloaded from the GEO database. Functional and pathway enrichment analysis of differentially expressed genes (DEGs) were performed and protein-protein interaction (PPI) network was analyzed using Cytoscape. In total, 999 DEGs were identified, including 667 up-regulated and 332 down-regulated DEGs. Gene ontology analysis demonstrated that most DEGs were significantly enriched in mitotic cell cycle, adhesion and protein binding process. Through PPI network analysis, a significant module was screened out, and the top 10 hub genes, CDK1, CCNB1, CENPE, CENPA, PLK1, CDC20, MAD2L1, HIST1H2BK, KIF2C and CCNA2 were identified from the PPI network. The expression levels of the 10 genes were validated in Oncomine database. KIF2C, MAD2L1 and PLK1 were associated with the overall survival. And we used cBioPortal to explore the genetic alterations of hub genes and potential drugs. In conclusion, the present study identified DEGs between normal and IDC samples, which could improve our understanding of the molecular mechanisms in the development of IDC, and these candidate genes might be used as therapeutic targets for IDC.

## INTRODUCTION

Breast cancer is the most common malignancy diagnosed in women worldwide, and is the second leading cause of cancer death among women. It has become one of the austerity issues in the world. Although the prognosis of patients is generally favorable due to the early detection and comprehensive treatment, the morbidity of breast cancer is rising. Additionally, the recurrence rate remains high and 20%–30% of patients will develop distant metastases with a median two-year survival time [1, 2]. Several studies have revealed that the rise of breast cancer cases is often due to the inherent susceptibility of genes and it is easy to relapse even after surgery of removing the primary tumor [3].

Invasive breast carcinoma is a heterogeneous group of tumors that exhibit different morphological spectrums and clinical behaviors. Treatment strategies for patients are designed based on the histological characteristics of tumor and other prognostic factors [4]. Breast cancer type is one of the most vital characteristics, and it is an important prognostic factor for breast cancer patients. To treat patients with invasive breast carcinoma, it is necessary for us to understand the specific biological characteristics of a given histological type [4]. According to a reported data, invasive ductal carcinoma (IDC) is a common histological type of breast cancer, accounting for about 75% of all invasive breast carcinoma cases [5]. Despite clinicians suggest that invasive ductal carcinoma always requires radical treatment, chemotherapy, and radiotherapy, to date, a lack of knowledge regarding the precise molecular targets for IDC limits the ability to treat advanced diseases [6].

Microarray is a high-throughput platform for the analysis of gene expression profiles, it has been widely used for investigating the underlying regulatory network involved in different types of cancer with great clinical applications: to improve the clinical diagnosis, and to discover new drug targets. Using microarray technology, several studies have exploited gene expression profiles of breast cancer and demonstrated prognostic significance. The analysis of BRCA1/2 mutation has been already used in clinical practice as a prognostic marker for breast cancer [7]. In a recent meta-analysis, AMDC2, TSHZ2, and CLDN11 were significantly related to the disease-free survival of breast cancer patients [6]. However, the results are often inconsistent due to sample heterogeneity.

In the present study, we have downloaded public microarray data from Gene Expression Omnibus (GEO, http://www.ncbi.nlm.nih.gov/geo/) to identify DEGs between IDC samples and normal samples. Subsequently, functions of DEGs were further analyzed by gene ontology (GO) annotation, pathway enrichment and protein-protein interaction (PPI) network construction using bioinformatics methods. By way of identifying DEGs and analyzing their biological functions and key pathways, we will have a better understanding of the mechanisms of IDC pathogenesis, and explore the potential candidate biomarkers for early diagnosis, individualize the prevention and therapy of IDC patients.

## RESULTS

### Identification of DEGs

In the present study, 143 IDC samples and 42 normal samples in the dataset of GSE10780 were analyzed. Based on the cut-off criteria (adjusted *P*-value < 0.01 and |log2 foldchange (FC)|> 1), a total of 999 DEGs were identified, including 667 up-regulated and 332 down-regulated DEGs. DEGs expression heat map is shown in Supplementary Figure 1, and the hierarchical cluster analysis of the data demonstrated that the DEGs could be used to distinguish IDC samples from the normal samples. We then analyzed GSE21422 to validate the results, the overlapping DEGs were identified using Venn diagram in Supplementary Figure 2.

### Gene ontology enrichment analysis

Functional enrichment analysis of DEGs was performed using DAVID online tool. The DEGs were categorized into three functional groups: biological process (BP), molecular function (MF) and cellular component (CC). GO analysis results (Table 1) showed that in the biological process, up-regulated genes were mainly enriched in cell division, sister chromatid cohesion, mitotic nuclear division, chromosome segregation and DNA replication; down-regulated genes were enriched in cell adhesion, hemidesmosome assembly, response to drug, and positive regulation of nitric oxide biosynthetic process. For molecular function, up-regulated genes were significantly enriched in protein binding, microtubule binding, protein kinase binding, identical protein binding and protein heterodimerization activity; while down-regulated genes were enriched in heparin binding, transcriptional activator activity, RNA polymerase II core promoter proximal region sequence-specific binding, growth factor activity, protein homodimerization activity and Wnt-protein binding. In the cellular component analysis, up-regulated genes were mainly enriched in nucleoplasm, condensed chromosome kinetochore, cytosol, chromosome, centromeric region and chromosome, centromeric region; down-regulated genes were enriched in extracellular space, extracellular region, proteinaceous extracellular matrix, extracellular exosome and cell surface. The results demonstrated that most DEGs were significantly enriched in mitotic cell cycle, adhesion and protein binding process.

**Table 1:** The top 5 enriched gene ontology terms of differentially expressed genes

Expression	Category	Term	Gene count	*P* value
Up-regulated	GOTERM_BP	GO:0051301~cell division	47	8.76E-15
	GOTERM_BP	GO:0007062~sister chromatid cohesion	24	8.39E-13
	GOTERM_BP	GO:0007067~mitotic nuclear division	36	1.94E-10
	GOTERM_BP	GO:0007059~chromosome segregation	18	1.22E-09
	GOTERM_BP	GO:0006260~DNA replication	24	4.72E08
	GOTERM_MF	GO:0005515~protein binding	378	6.99E-09
	GOTERM_MF	GO:0008017~microtubule binding	20	1.44E-04
	GOTERM_MF	GO:0019901~protein kinase binding	29	1.65E-04
	GOTERM_MF	GO:0042802~identical protein binding	47	1.76E-04
	GOTERM_MF	GO:0046982~protein heterodimerization activity	33	2.47E-04
	GOTERM_CC	GO:0005654~nucleoplasm	161	2.31E-12
	GOTERM_CC	GO:0000777~condensed chromosome kinetochore	20	7.16E-11
	GOTERM_CC	GO:0005829~cytosol	173	1.21E-09
	GOTERM_CC	GO:0000775~chromosome, centromeric region	15	4.51E-09
	GOTERM_CC	GO:0070062~extracellular exosome	144	1.80E-07
Down-regulated	GOTERM_BP	GO:0007155~cell adhesion	22	2.10E-05
	GOTERM_BP	GO:0031581~hemidesmosome assembly	5	3.05E-05
	GOTERM_BP	GO:0042493~response to drug	17	3.99E-05
	GOTERM_BP	GO:0045429~positive regulation of nitric oxide biosynthetic process	7	6.43E-05
	GOTERM_BP	GO:0007568~aging	12	8.28E-05
	GOTERM_MF	GO:0008201~heparin binding	14	1.00E-06
	GOTERM_MF	GO:0001077~transcriptional activator activity, RNA polymerase II core promoter proximal region sequence-specific binding	15	1.54E05
	GOTERM_MF	GO:0008083~growth factor activity	11	1.85E-04
	GOTERM_MF	GO:0042803~protein homodimerization activity	25	3.21E-04
	GOTERM_MF	GO:0017147~Wnt-protein binding	5	0.001
	GOTERM_CC	GO:0005615~extracellular space	62	1.12E-13
	GOTERM_CC	GO:0005576~extracellular region	62	2.10E-10
	GOTERM_CC	GO:0005578~proteinaceous extracellular matrix	20	7.82E-08
	GOTERM_CC	GO:0070062~extracellular exosome	72	5.55E-05
	GOTERM_CC	GO:0009986~cell surface	23	7.34E-05

*BP, biological process; MF, molecular function; CC, cellular component.

*P* value < 0.01 was considered as threshold values of significant difference.

### Pathway enrichment analysis

According to KEGG pathway analysis, significantly enriched pathways of DEGs were shown in Table 2. Up-regulate genes were mainly enriched in cell cycle, DNA replication, viral carcinogenesis, systemic lupus erythematosus and pyrimidine metabolism pathways; while down-regulate genes were significantly enriched in regulation of lipolysis in adipocytes, PI3K-Akt signaling pathway, PPAR signaling pathway and cytokine-cytokine receptor interaction pathways.

**Table 2:** KEGG pathway analysis of differentially expressed genes

Expression	Pathway	Gene count	*P* value
Up-regulated	hsa04110: Cell cycle	22	3.75E-07
	hsa03030: DNA replication	9	6.68E-05
	hsa05203: Viral carcinogenesis	21	1.84E-04
	hsa05322: Systemic lupus erythematosus	16	2.71E-04
	hsa00240: Pyrimidine metabolism	13	8.41E-04
	hsa04114: Oocyte meiosis	12	0.004
	hsa05034: Alcoholism	16	0.005
	hsa05161: Hepatitis B	14	0.005
	hsa03410: Base excision repair	6	0.009
Down-regulated	hsa04923: Regulation of lipolysis in adipocytes	7	4.81E-04
	hsa04151: PI3K-Akt signaling pathway	15	0.004
	hsa03320: PPAR signaling pathway	6	0.007
	hsa04060: Cytokine-cytokine receptor interaction	11	0.008

*P* value < 0.01 was considered as threshold values of significant difference.

### PPI network analysis

The 999 DEGs were submitted to the STRING database to predict the protein interactions. With combined score greater than 0.7, the PPI network consisted of 955 nodes and 2246 edges. The top 10 hub nodes with higher degrees were screened out using the plug-in CytoHubba in Cytoscape. These hub genes included cyclin-dependent kinase 1 (CDK1), cyclin B1 (CCNB1), centromere protein E (CENPE), centromere protein A (CENPA), polo-like kinases 1 (PLK1), cell division cycle 20 (CDC20), MAD2 mitotic arrest deficient-like 1 (MAD2L1), histone cluster 1, H2bk (HIST1H2BK), kinesin family member 2C (KIF2C) and cyclin A2 (CCNA2).

**Figure 1:**
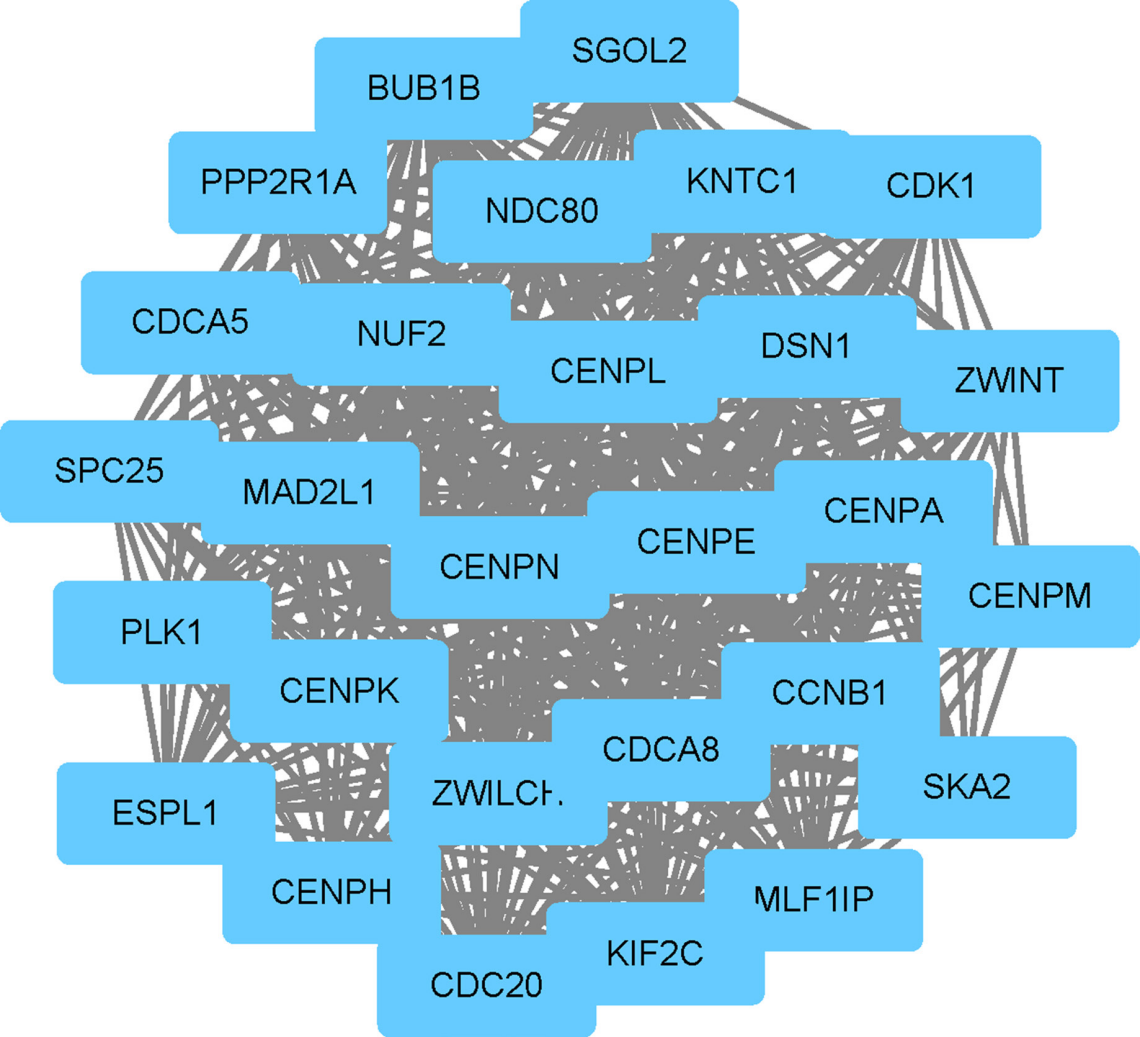
The significant module identified from the protein-protein interaction network

Moreover, the total of 955 nodes and 2246 edges were analyzed using plug-in MCODE, and the most significant modules were screened out, which contained 13 nodes and 75 edges (Figure 1). Strikingly, all of the genes in this module were up-regulated DEGs. According to GO enrichment analysis, in biological process, the genes were mainly associated with sister chromatid cohesion, cell division, mitotic nuclear division, mitotic sister chromatid segregation and chromosome segregation. In molecular function, these genes were significantly enriched in protein binding, histone kinase activity, kinetochore binding, anaphase-promoting complex binding and centromeric DNA binding. In the cellular component analysis, they were mainly enriched in condensed chromosome kinetochore, cytosol, chromosome, centromeric region, kinetochore and spindle pole (Table 3). KEGG pathway analysis demonstrated that these genes were mainly involved in cell cycle, oocyte meiosis and progesterone-mediated oocyte maturation pathways (Table 4).

**Table 3:** The top 5 enriched gene ontology terms the significant module

Category	Term	Gene count	*P* value
GOTERM_BP	GO:0007062~sister chromatid cohesion	22	4.90E-44
GOTERM_BP	GO:0051301~cell division	17	1.54E-21
GOTERM_BP	GO:0007067~mitotic nuclear division	13	3.57E-16
GOTERM_BP	GO:0000070~mitotic sister chromatid segregation	7	9.90E12
GOTERM_BP	GO:0007059~chromosome segregation	8	4.43E-09
GOTERM_MF	GO:0005515~protein binding	24	2.95E-07
GOTERM_MF	GO:0035173~histone kinase activity	2	0.005
GOTERM_MF	GO:0043515~kinetochore binding	2	0.005
GOTERM_MF	GO:0010997~anaphase-promoting complex binding	2	0.008
GOTERM_MF	GO:0019237~centromeric DNA binding	2	0.009
GOTERM_CC	GO:0000777~condensed chromosome kinetochore	14	1.30E-24
GOTERM_CC	GO:0005829~cytosol	26	2.91E-19
GOTERM_CC	GO:0000775~chromosome, centromeric region	10	2.89E-17
GOTERM_CC	GO:0000776~kinetochore	9	1.09E-13
GOTERM_CC	GO:0000922~spindle pole	6	3.37E-07

BP, biological process; MF, molecular function; CC, cellular component.

*P* value < 0.01 was considered as threshold values of significant difference.

**Table 4:** KEGG pathway analysis of the significant module

Pathway	Gene count	*P* value
hsa04110: Cell cycle	7	8.05E-10
hsa04114: Oocyte meiosis	6	4.81E-08
hsa04914: Progesterone-mediated oocyte maturation	4	1.03E-04

*P* value < 0.01 was considered as threshold values of significant difference

### Validation of the hub genes

**Figure 2:**
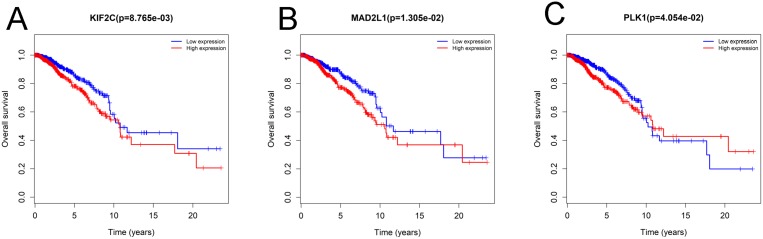
Three hub genes were associated with overall survival in invasive ductal carcinoma patients by using Kaplan-Meier curve and log-rank test The patients were stratified into high level group and low level group according to median of each gene. (**A**) KIF2C. (**B**) MAD2L1. (**C**) PLK1.

**Figure 3:**
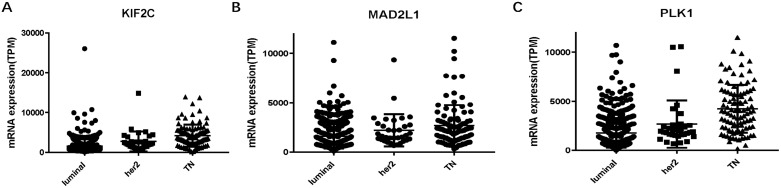
L KIF2C, MAD2L and PLK1 expressions among molecular subtypes of breast cancer (**A**) KIF2C. (**B**) MAD2L1. (**C**) PLK1. *TN: triple negative.*

To confirm the reliability of the hub genes, we used ONCOMINE (www.oncomine.org), a cancer microarray database and web-based data-mining platform to validate the expression levels of the 10 genes [8]. We performed the differential analysis between IDC and normal samples using TGCA datasets. Consistently, the top 10 hub genes were significantly up-regulated in IDC (Supplementary Figure 3). In order to identify the hub genes which would be potentially associated with overall survival of IDC patients, we evaluated the associations between hub genes’ expression and patients’ survival using Kaplan-Meier curve and Log-rank test. The results showed that 3 hub genes (KIF2C, MAD2L1 and PLK1) were associated with the overall survival (Figure 2). We summarized the association of the three hub genes’ expression levels and clinical features in Tables 5–7. The result demonstrated that MAD2L1and KIF2C were significantly associated with patients age, ER and PR status, tumor stage and size; PLK1was related to ER and PR status, tumor stage and size. The difference expressions of MAD2L, KIF2C and PLK1 among molecular subtypes were shown in Figure 3.

**Table 5:** Association of MAD2L1 expression level and clinical features

Variables	Low expression	High expression	*P* value^*^
**Age at diagnosis, y**			0.041
**< 35**	9	17	
**35-49**	92	108	
**50-64**	162	169	
**≥65**	162	95	
**Sex**			0.362
**Male**	385	382	
**Female**	4	7	
**ER**			*P* < 0.001
**Negative**	67	136	
**Positive**	307	231	
**Equivocal**	5	4	
**Unknown**	10	18	
**PR**			*P* < 0.001
**Negative**	100	179	
**Positive**	272	188	
**Equivocal**	6	5	
**Unknown**	11	17	
**HER2**			0.324
**Negative**	207	286	
**Positive**	62	65	
**Equivocal**	55	72	
**Unknown**	65	66	
**Stage**			0.001
**I**	90	49	
**II**	211	241	
**III**	68	86	
**IV**	11	6	
**Unknown**	9	7	
**Tumor size**			*P* < 0.001
**T1**	137	82	
**T2**	212	255	
**T3**	21	37	
**T4**	18	14	
**Unknown**	1	1	
**Lymph node stage**			0.243
**N0**	185	172	
**N1**	134	141	
**N2**	39	55	
**N3**	22	16	
**Unknown**	9	5	
**Distant metastasis**			0.204
**M0**	328	345	
**M1**	11	8	
**Unknown**	50	36	

*P values calculated by Pearson Chi squared or Fisher's exact testing.

y: years.

**Table 6:** Association of KIF2C expression level and clinical features

Variables	Low expression	High expression	*P* value^*^
**Age at diagnosis, y**			0.022
**<35**	9	17	
**35-49**	92	108	
**50-64**	160	71	
**≥65**	128	93	
**Sex**			0.362
**Male**	385	382	
**Female**	4	7	
**ER**			*P* < 0.001
**Negative**	42	161	
**Positive**	331	207	
**Equivocal**	4	5	
**Unknown**	12	16	
**PR**			*P* < 0.001
**Negative**	78	201	
**Positive**	294	166	
**Equivocal**	5	6	
**Unknown**	12	16	
**HER2**			0.3870
**Negative**	199	194	
**Positive**	60	67	
**Equivocal**	66	61	
**Unknown**	64	67	
**Stage**			0.001
**I**	92	47	
**II**	212	240	
**III**	69	85	
**IV**	8	9	
**Unknown**	8	8	
**Tumor size**			*P* < 0.001
**T1**	142	77	
**T2**	214	253	
**T3**	16	42	
**T4**	16	16	
**Unknown**	1	1	
**Lymph node stage**			0.378
**N0**	184	173	
**N1**	138	137	
**N2**	39	55	
**N3**	19	19	
**Unknown**	9	5	
**Distant metastasis**			0.204
**M0**	332	341	
**M1**	8	11	
**Unknown**	49	37	

*P values calculated by Pearson Chi squared or Fisher's exact testing.

y: years.

**Table 7:** Association of PLK1 expression level and clinical features

Variables	Low expression	High expression	*P* value^*^
**Age at diagnosis, y**			0.587
**< 35**	12	14	
**35–49**	96	104	
**50–64**	162	169	
**≥65**	119	102	
**Sex**			0.761
**Male**	384	383	
**Female**	5	6	
**ER**			*P* < 0.001
**Negative**	48	155	
**Positive**	329	209	
**Equivocal**	3	6	
**Unknown**	9	19	
**PR**			*P* < 0.001
**Negative**	83	196	
**Positive**	293	167	
**Equivocal**	3	8	
**Unknown**	10	18	
**HER2**			0.452
**Negative**	206	187	
**Positive**	57	70	
**Equivocal**	64	63	
**Unknown**	62	69	
**Stage**			*P* < 0.001
**I**	98	41	
**II**	208	244	
**III**	67	87	
**IV**	7	10	
**Unknown**	9	7	
**Tumor size**			*P* < 0.001
**T1**	150	69	
**T2**	208	259	
**T3**	15	43	
**T4**	15	17	
**Unknown**	1	1	
**Lymph node stage**			0.213
**N0**	183	174	
**N1**	140	135	
**N2**	37	57	
**N3**	20	18	
**Unknown**	9	5	
**Distant metastasis**			0.284
**M0**	334	339	
**M1**	7	12	
**Unknown**	48	38	

*P values calculated by Pearson Chi squared or Fisher's exact testing.

y: years.

### Mining genetic alterations and potential drugs of hub genes

**Figure 4:**
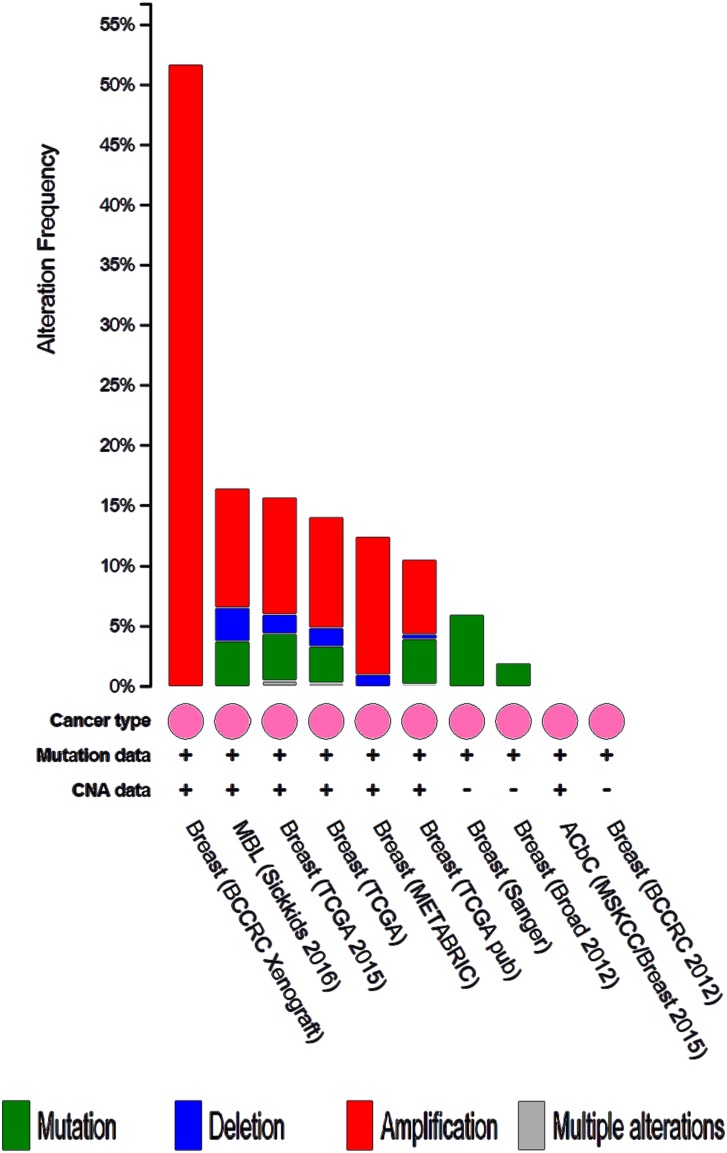
Summary of alterations for CDK1, CCNB1, CENPE, CENPA, PLK1, CDC20, MAD2L1, HIST1H2BK, KIF2C and CCNA2 in breast cancer

**Figure 5:**
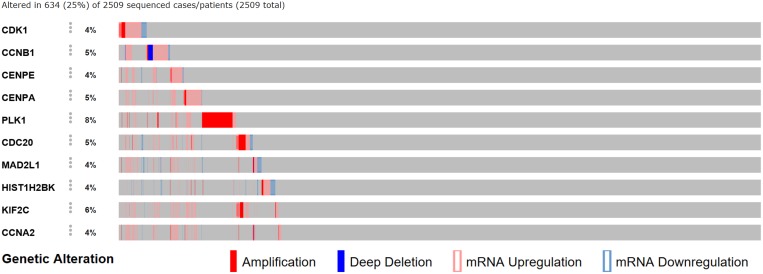
A visual summary of alteration across a set of breast samples (data taken from the study of Curtis et al and/or Pereira et al.) based on a query of the hub genes.

**Figure 6:**
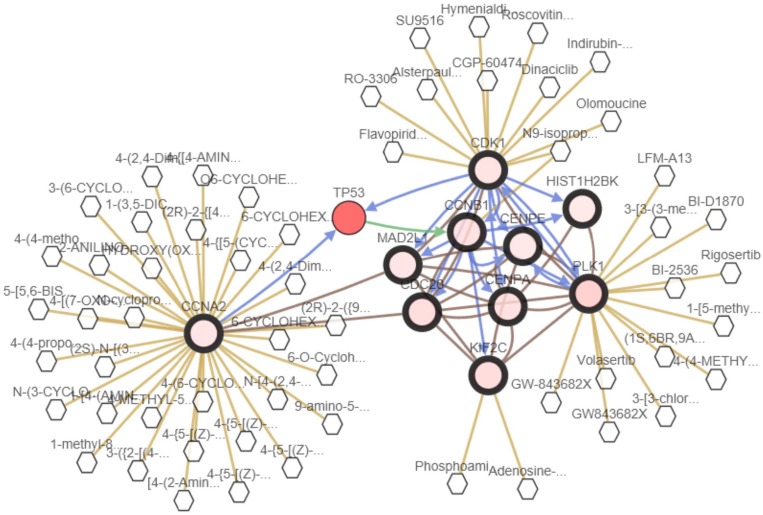
A visual display of the drugs connected to CDK1, CCNB1, CENPE, CENPA, PLK1, CDC20, MAD2L1, HIST1H2BK, KIF2C and CCNA2 in breast cancer (based on the study of Curtis et al. and/or Pereira et al.)

We analyzed the 10 hub genes using cBioportal to explore their cancer genomic alterations in breast cancer and to find out potential drugs. Among the 10 breast cancer studies, alterations ranging for the hub genes were found from 0% to 51.7% (Figure 4). In the study of Curtis et al. and/or Pereira et al. [9], 634 cases (25%) had an alteration in at least one of the 10 genes queried. The frequency of alteration in each of the selected genes was shown in Figure 5. Mutual exclusivity analysis showed that 43 gene pairs had significant co-occurrent alterations (Supplementary Table 1). The drugs for hub genes were showed in Figure 6. Among them, CCNA2, CDK1 and PLK1 were targets of most drugs.

## DISCUSSION

IDC is a common histological type of breast cancer, it is important to understand the molecular mechanisms for the treatments. Nowadays, microarray has been widely used to analyze the expression changes of mRNA in breast cancer and predict the potential therapeutic targets. In the present study, we explored potential crucial genes associated with IDC using bioinformatics analysis. Based on the cutoff criteria, a total of 667 up-regulated and 332 down-regulated DEGs were identified from GSE10780.

Through GO enrichment analysis, in biological process, the up-regulated genes were most significantly involved in cell division. While down-regulated genes were significantly enriched in cell adhesion. It is easy to understand that uncontrolled cell division is the hallmark of cancers, and loss of cell-cell adhesion is an important step in the acquisition of the invasive, metastatic phenotype [10, 11]. In the molecular function portion, up-regulated genes were mainly enriched in protein binding, microtubule binding and protein kinase binding. It is pointed out that many microtubule binding proteins were associated with oncogenesis. Microtubule end-binding protein 1 (EB1) was demonstrated up-regulated both in human breast cancer specimens and cell lines. The level of EB1 could indicate the malignancy of breast cancer and is reported to be correlated with clinical characteristics, including higher histological grade, higher pathological tumor node metastasis stage (pTNM), and higher incidence of lymph node metastasis [12]. While down-regulated genes were most significantly enriched in heparin binding, a previous study reported that the expression of heparin-binding epidermal growth factor-like growth factor (HB-EGF) was inversely related to biological aggressiveness of the breast carcinoma, suggesting that HB-EGF may play an important role in process of breast carcinoma [13]. As for cellular component, up-regulated genes mainly located in the cell nucleus, and down-regulated genes were mostly enriched in extracellular positions. This result indicated that DEGs may participate in DNA replication and cell adhesion. GO enrichment analysis demonstrated that DEGs might play crucial roles in oncogenesis through cell division, adhesion and binding-related mechanisms.

Furthermore, pathway analysis revealed that up-regulated genes were mainly engaged in cell cycle and DNA replication, suggesting DEGs may participate in cell proliferation. Down-regulated genes were enriched in regulation of lipolysis in adipocytes, PI3K-Akt signaling pathway, PPAR signaling pathway and cytokine-cytokine receptor interaction. Recent studies have demonstrated that obesity is associated with increased recurrence and reduced survival of breast cancer, and adipocyte lipolysis may play an important role in the provision of metabolic substrates to breast cancer cells. While further studies are needed to explore the complex metabolic symbiosis between tumor-surrounding adipocytes and cancer cells that stimulate their invasiveness [13, 14]. In addition, PI3K-Akt signaling pathway and PPAR signaling pathway were confirmed that both play crucial roles in cell proliferation, invasion and metastasis [15], PI3K-Akt signaling pathway had been demonstrated to be activated in breast cancer, in the present study, 15 genes (IL6, FGF10, GNG11, KIT, PCK1, LAMB3, COL6A6, RELN, LAMC2, ANGPT1, TNN, PDGFD, EGF, PIK3R1, GHR) were down regulated in this pathway, and the result was validated using TCGA database. These genes are not crucial genes in the PI3K-Akt signaling pathway, they may participate other pathways which can regulate tumorigenesis, the downregulation of these genes may promote the occurrence and development of tumor.

Through PPI network construction, the module analysis revealed that the DEGs were mainly involved in cell division, cycle and binding-related mechanisms. And we listed the top 10 hub genes with higher degrees: CDK1, CCNB1, CENPE, CENPA, PLK1, CDC20, MAD2L1, HIST1H2BK, KIF2C and CCNA2. The 10 hub genes were validated in the TCGA database. PLK1, MAD2L1 and KIF2C were demonstrated significantly associated with overall survival and clinical features. In addition, these hub genes all had alterations in breast cancer.

CDK1 have been demonstrated to be a potential prognostic indicator, the protein encoded by this gene is a member of the Ser/Thr protein kinase family, which is essential for G1/S and G2/M phase transitions of eukaryotic cell cycle. S. J. Kim et al. considered that CDK1 is strongly associated with clinical outcomes of breast cancer patients, and regarded CDK1 as a new independent prognostic factor [16, 17]. CCNB1 is a regulatory protein involved in mitosis, it is expressed predominantly during G2/M phase. It is reported that CCNB1 is a power prognostic factor for the survival of ER+ breast cancer patients [18], and it is also involved in therapy resistance [19]. CENPE is a kinesin-like motor protein that accumulates in the G2 phase of the cell cycle, while CENPA is proposed to be a component of a modified nucleosome or nucleosome-like structure. CENPE and CENPA are two AU-rich elements (AREs) involved in the mitotic cell cycle, a recent study revealed that defects in ARE-mediated posttranscriptional control could lead to carcinogenesis. Recent studies have also shown that the survival of breast cancer patients is related to high levels of the mitotic ARE-mRNA signature [20]. PLK1 is a Ser/Thr protein kinase which performs important roles in the M phase of the cell cycle. PLK1 antagonizes p53 during DNA damage response, and alteration of mRNA and protein expression related to DNA damaging, replication and repairing was detected in PLK1-silenced tumor cells, including the DNA-dependent protein kinase (DNAPK) and topoisomerase II alpha (TOPO2A) [21]. M Wierer et al. reported PLK1 mediates estrogen receptor (ER)-regulated gene transcription in human breast cancer cells [22]. Evidence also revealed that breast cancer cells with treatment of siRNAs targeting PLK1 could improve the sensitivity toward paclitaxel and Herceptin [23]. CDC20 acts as a regulatory protein which is an essential component of cell division. High CDC20 is reported to be associated with an aggressive course of disease and poor prognosis [24]. MAD2L1 is a component of the mitotic spindle assembly checkpoint, and it may play crucial roles in the progression of breast cancer. Interestingly, MAD2L1 could inhibit the activity of the anaphase promoting complex by sequestering CDC20 until all chromosomes are aligned at the metaphase plate. Lowering the expression of MAD2L1 by siRNAs could reduce tumor cell growth and inhibit cell migration and invasion [25]. MAD2 overexpression has recently been shown to lead to tumor initiation and progression through the acquisition of chromosomal instability (CIN) in mice, tumors that experience transient MAD2 overexpression and consequent CIN results in markedly elevated recurrence rates [26]. It is reported that measuring the expression of MAD2L1 may assist the prediction of breast cancer prognosis [25]. The potential mechanisms of MAD2L1 in breast cancer require further investigation. HIST1H2BK is a core component of nucleosome, which participates in the pathway of activated PKN1 stimulates transcription of androgen receptor regulated genes KLK2 and KLK3. While the role of HIST1H2BK in breast cancer remains unclear. KIF2C is a kinesin-like protein that functions as a microtubule-dependent molecular motor. It can depolymerize microtubules at the plus end, thereby promoting mitotic chromosome segregation. T Abdelfatah et al. revealed the overexpression of KIF2C protein is associated with unfavorable clinic-pathological features and predicted poor clinical outcomes [27]. While the underlying mechanisms are not clear, further investigations are needed to identify the role of KIF2C in breast cancer. CCNA2 functions as a regulator of the cell cycle to promote transition through G1/S and G2/M. Several studies have demonstrated that CCNA2 has significant power to predict the survival of breast cancer patients and it is also found that CCNA2 was closely associated with tamoxifen resistance [28, 29]. In our present study, only MAD2L1, KIF2C and PLK1 were associated the overall survival of IDC patients. The three genes all play important roles in the process of mitotic cell cycle. It is easy to understand that uncontrolled cell cycle is an important step of cancer occurrence and development, while further studies are still required to explore the mechanisms.

Using bioinformatics analysis, our study identified 667 up-regulated and 332 down-regulated DEGs. Among the10 hub genes, MAD2L1, KIF2C and PLK1 were potential biomarkers for the prognosis of IDC patients. The results of the present study may give valuable indication for basic and clinical research. However, further molecular biological experiments are needed in order to confirm the functions of identified DEGs.

## MATERIALS AND METHODS

### Identification of DEFs in IDC

The gene expression profiles of GSE10780 were downloaded from the GEO database. GSE10780 which was submitted by Chen D et al. was based on GPL570 platform (Affymetrix Human Genome U133 Plus 2.0 Array) and contained 185 samples, including 42 histologically normal breast tissues and 143 IDC tissues. The probes without annotation of gene expression profiles were filtered and probes were transformed into gene symbol. The gene expression profile data was preprocessed using the Robust Multi-array Average (RMA) algorithmin affy package within Bioconductor (http://www.bioconductor.org) in R. After background correction, quantile normalization and probe summarization, we used the Linear Models for Microarray Data (LIMMA, http://www.bioconductor.org/packages/release/bioc/html/limma.html) package in R to identify DEGs by comparing expression value between samples in IDC and normal group. The corresponding *p* value of gene symbols after classical *T*-test was defined as adjusted *p*-value, adjusted *P*-value <0.01 and |log2 foldchange (FC)|>1 were set as the cutoff criteria. And five healthy tissue samples and five IDC samples from GSE21422 (based on GPL570 platform) were analyzed to validate the results.

### Gene ontology and pathway enrichment analysis of DEGs

Functional and pathway enrichment analysis of DEG were carried out using the database for Annotation, Visualization and Integrated Discovery (DAVID, http://david.abcc.ncifcrf.gov/). In the present study, we performed Gene Ontology (GO) and Kyoto Encyclopedia of Genes and Genomes (KEGG) pathway analysis based on DAVID online tools. The Go terms were classified into three categories, including cellular component (CC), biological process (BP) and molecular function (MF). *P*-value < 0.01 was considered as statistically significant differences. For KEGG pathway analysis, *P*-value < 0.01 was set as the cut-off criterion to identify the enriched pathways.

### Construction of PPI network

The PPI network of DEGs in our study were constructed using Search Tool for the Retrieval of Interacting Gene (STRING) database. STRING is an online tool to predict the protein-protein interaction information, and can provide system-wide view of cellular processes. To evaluate the interactive relationships among DEGs, we established the PPI network using STRING, and “Confidence score > 0.7” was selected as the cut-off criterion. Then, PPI network was visualized by cytoscape software (http://www.cytoscape.org/). Molecular Complex Detection (MCODE) was subsequently applied to screen the modules of PPI network in cytoscape. The criteria were set as follows: “degree cutoff = 2”, “node score cutoff = 0.2”, “k-core = 2” and “max.depth = 100”. The hub proteins are a small number of proteins that have many interactions with other proteins, to screen out these important nodes in the PPI network, the plug-in CytoHubba was utilized in the present study.

### Validation of the hub genes

To confirm the reliability of the hub genes, we used ONCOMINE (www.oncomine.org), a cancer microarray database and web-based data-mining platform to validate the expression levels of the 10 genes [8]. We performed differential analysis between IDC and normal samples using TGCA datasets. In addition, the RNA sequencing data and clinical information were downloaded from TCGA database (https://cancergenome.nih.gov/). A total of 778 IDC cases were analyzed in our study. The RNA sequencing data were normalized using R language package. Patients clinical information included sex (male and female), age at diagnosis (<35, 35-49, 50-64, ≥65 years), race (white, black, Asian), ER, PR and HER2 status, and tumor-node-metastasis (TNM) stage. The prognostic value of each differentially expressed mRNA was evaluated using Kaplan -Meier survival curves by log-rank test. A *p*-value < 0.05 was defined as significant.

### Exploring cancer genomics data by cBioportal

The cBioPortal for Cancer Genomics (http://www.cbioportal.org/) provides visualization, analysis and download of large-scale cancer genomics data sets [30, 31]. In this study, we used cBioPortal to explore the genetic alterations of hub genes and potential drugs.

### Human participants and animal rights

This article does not contain any studies with human participants or animals performed by any of the authors.

## SUPPLEMENTARY MATERIALS FIGURES AND TABLES




