# Supplementary material for: Identification of the potential crucial genes in invasive ductal carcinoma using bioinformatics analysis
Source: Oncotarget. 2017 Dec 13;9(6):6800–13. doi: 10.18632/oncotarget.23239 (PMC5805516; doi:10.18632/oncotarget.23239)
Supplement: Supplementary file 1 [file oncotarget-09-6800-s001.pdf]

Identification of the potential crucial genes in invasive ductal carcinoma using bioinformatics analysis

SUPPLEMENTARY MATERIALS

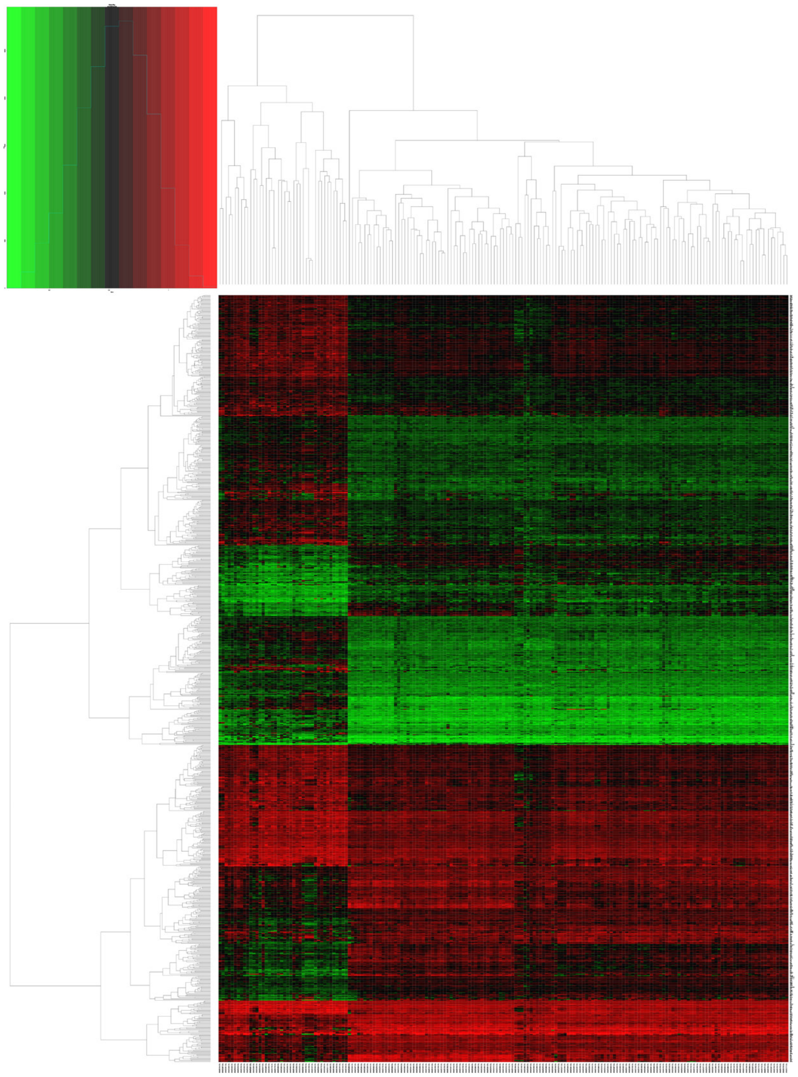

Supplementary Figure 1: Heat map of the differentially expressed genes.

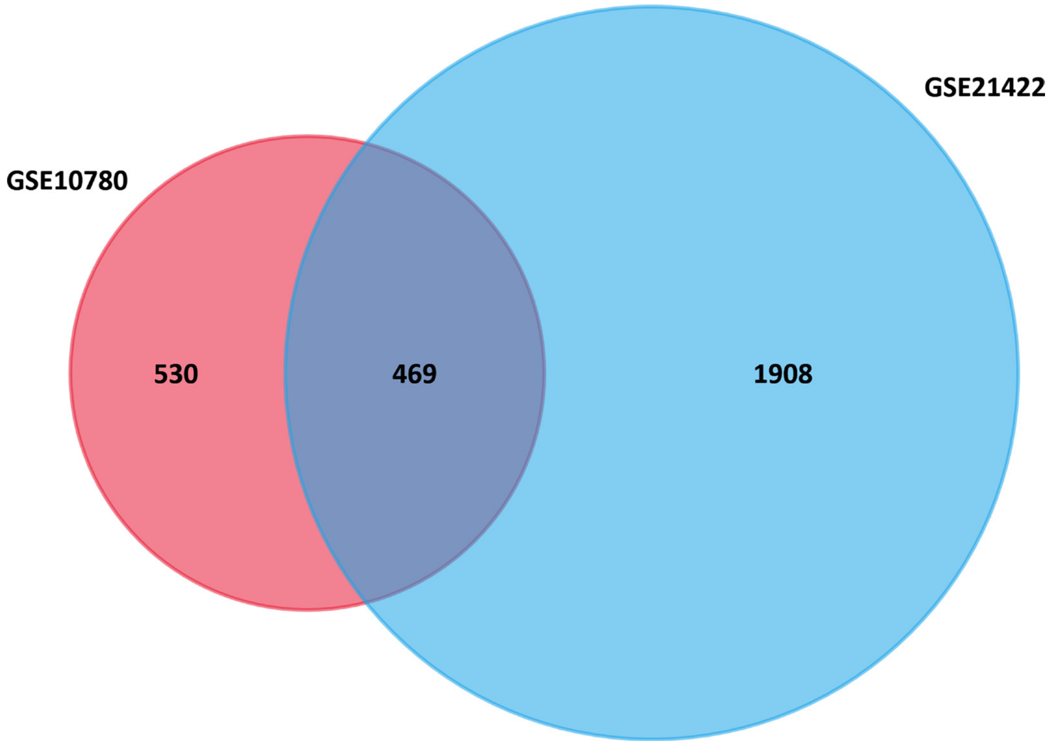

Supplementary Figure 2: The overlapping genes of GSE10780 and GSE21422.

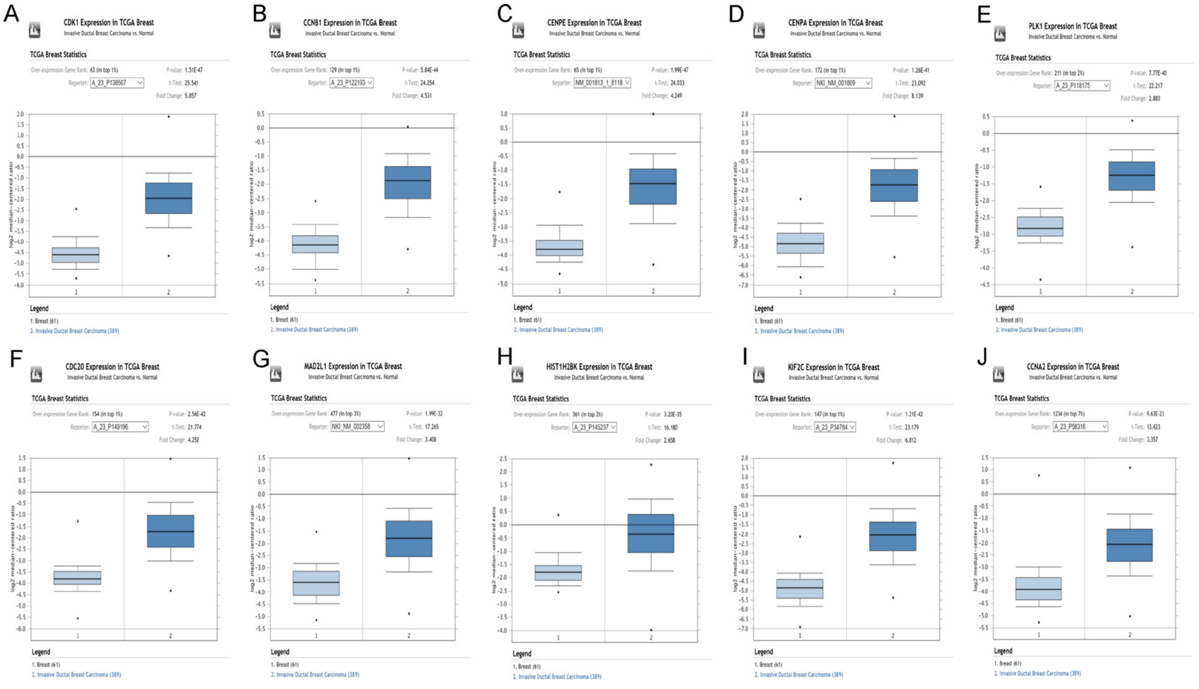

Supplementary Figure 3: The expression of the 10 hub genes in TGCA. (**A**). The expression of CDK1. (**B**). The expression of CCNB1. (**C**). The expression of CENPE. (**D**). The expression of CENPA. (**E**). The expression of PLK1. (**F**). The expression of CDC20. (**G**). The expression of MAD2L1. (**H**). The expression of HIST1HIBK. (**I**). The expression of KIF2C. (**J**). The expression of CCNA2.

Supplementary Table 1: Mutual exclusivity analysis of hub genes. See_Supplementary_Table 1
